# Design and rationale of FOCUS (PX-171-011): A randomized, open-label, phase 3 study of carfilzomib versus best supportive care regimen in patients with relapsed and refractory multiple myeloma (R/R MM)

**DOI:** 10.1186/1471-2407-12-415

**Published:** 2012-09-19

**Authors:** Roman Hájek, Richard Bryce, Sunhee Ro, Barbara Klencke, Heinz Ludwig

**Affiliations:** 1 University Hospital Brno and Faculty of Medicine Ostrava, Department of Internal Medicine -Haematooncology, Jihlavska 20, Brno, 625 00, Czech Republic; 2 Onyx Pharmaceuticals, Inc, South San Francisco, CA, USA; 3 Wilhelminen Hospital, Vienna, Austria; 4 Present affiliation: Puma Biotechnology, Inc, Los Angeles, CA, USA

**Keywords:** Multiple myeloma, Proteasome inhibitor, Phase 3 trial, Relapsed, Refractory, Overall survival

## Abstract

**Background:**

Carfilzomib is a next-generation proteasome inhibitor with single-agent activity in patients with relapsed and refractory multiple myeloma (R/R MM). In PX-171-003-A1, a single-arm phase 2 study of carfilzomib monotherapy in heavily pretreated patients, the overall response rate was 23.7%, 37% of patients achieved ≥ minimal response and median overall survival (OS) was 15.6 months. Based on this study, carfilzomib was recently approved by the US Food and Drug Administration for the treatment of R/R MM. Herein we describe the trial design and rationale for a phase 3 randomized study, FOCUS (CarFilzOmib for AdvanCed Refractory MUltiple Myeloma European Study), being conducted to compare OS after treatment with single-agent carfilzomib to best supportive care (BSC) regimen in R/R MM.

**Methods:**

Patients must have received ≥3 prior regimens, must be responsive to at least 1 line of therapy, and be refractory to their most recent therapy. Eligible patients are randomized 1:1 to receive either carfilzomib (28-day cycles at 20 mg/m^2^ IV on Days 1–2 of Cycle 1, escalating to 27 mg/m^2^ IV on Days 8, 9, 15, and 16 and continuing at 27 mg/m^2^ through Cycle 9 and Days 1, 2, 15, and 16 ≥ Cycle 10) or an active BSC regimen (corticosteroid treatment of prednisolone 30 mg, dexamethasone 6 mg, or equivalent every other day with optional cyclophosphamide 50 mg PO once daily). Patients will continue treatment until disease progression, unacceptable toxicity, or treatment discontinuation and will then enter long-term follow-up for survival. The primary endpoint is OS and secondary endpoints include progression-free survival, overall response rate, and safety. Disease assessments will be determined according to the International Myeloma Working Group Uniform Response Criteria with minimal response per European Blood and Marrow Transplantation Group criteria.

**Conclusions:**

This phase 3 trial will provide more rigorous data for carfilzomib, as this is the first carfilzomib study with OS as the primary endpoint and will not be confounded by crossover and will provide more robust secondary response and safety results that will add to the data set from prior phase 2 studies. FOCUS will facilitate regulatory approvals around the world and expand treatment options for patients with R/R MM.

**Trial registration:**

EudraCT No. 2009-016840-38; NCT01302392.

## Background

Multiple myeloma (MM) is the second most common hematologic malignancy worldwide [1]. Approximately 86,000 cases occur annually worldwide, with the frequency very unevenly distributed and the highest incidence in industrialized areas of Australia/New Zealand, Europe, and North America [2]. In Europe, MM has an incidence of 6/100,000 people and is responsible for at least 19,000 deaths a year [1,3,4]. MM is characterized by accumulation of abnormal plasma cells in bone marrow and monoclonal protein in blood and urine and has a heterogeneous clinical presentation [1,4]. Monoclonal gammopathy of undetermined significance (MGUS) consistently precedes development of MM [1].

Although there are MM treatments, the disease remains incurable and survival is limited to 6 to 7 years after autologous stem cell transplantation (ASCT) for patients who undergo the procedure and approximately 3 years in patients who do not [1,5,6]. Current treatments for relapsed and/or refractory disease include combination regimens using melphalan or alkylating agents, bortezomib, thalidomide, and lenalidomide with or without corticosteroids [6]. New therapies in various treatment settings have substantially improved outcomes for patients with relapsed and/or refractory disease, although survival after treatment with all available regimens, including those containing novel agents, is approximately 9 months following becoming refractory to these drugs [7]. Best supportive care (BSC) for patients with advanced refractory disease for which no approved alternative treatment is available may include low-dose glucocorticoids and/or low-dose alkylating agents, along with other supportive and palliative care options [8].

Carfilzomib is a next-generation proteasome inhibitor that is structurally and mechanistically distinct from bortezomib. It selectively and irreversibly inhibits the chymotrypsin-like activities of both the constitutive proteasome and the immunoproteasome [9,10]. In preclinical studies, carfilzomib displayed potent cytotoxic and pro-apoptotic activity across a broad panel of tumor-derived cell lines; was a potent proteasome inhibitor in early rodent tissue studies; showed potent inhibition of the proteasome with rapid and irreversible target binding despite high systemic clearance in rats; and was well tolerated in rats and monkeys [11-13].

Early phase 1 studies of carfilzomib showed sustained proteasome inhibition [14,15] and an acceptable safety profile in addition to establishing activity and efficacy in patients with MM [15]. Observation of fever, chills, shortness of breath, and/or rigors in these studies led to a selection of 20 mg/m^2^ as the initial dose, as well as inclusion of hydration and concomitant dexamethasone (4-mg predose during the first cycle). Further investigation subsequently showed higher doses of carfilzomib to be effective, permitting escalated dosing up to 27 mg/m^2^ beginning with Cycle 2 [15-18].

Carfilzomib has shown promising activity and tolerability in several phase 2 clinical studies [16]. In PX-171-003-A1, a large single-arm study of carfilzomib monotherapy in heavily pretreated patients with MM, the overall response rate (ORR) was 23.7% and 37% of patients achieved at least a minimal response (MR) [17]. The duration of response (DOR) was 7.8 months, and overall survival (OS) was 15.6 months [17]. In a study of bortezomib-treated and bortezomib-naïve patients with relapsed and/or refractory MM, PX-171-004, overall response rates (ORR) and clinical benefit response (CBR) of 17.1% and 31.4% were reported for bortezomib treated patients [19] and 47.6% and 61.9% were reported for bortezomib-naïve patients [20]. Additionally, carfilzomib has been investigated in patients with varying degrees of renal insufficiency (PX-171-005), and an ORR of 21.3% was observed with no difference in carfilzomib pharmacokinetics detected between the cohorts of patients with normal, mild, and severe renal dysfunction and no indication that a change in dose or treatment schedule is necessary for these patients [21]. Tolerability of extended treatment duration has been shown in long-term follow-up of PX-171-003-A1 [22].

A cross-trial safety analysis of 768 patients, including 526 patients from the four phase 2 trials 003-A0, 003-A1, 004, and 005 revealed an acceptable safety profile for carfilzomib [23,24]. Importantly, carfilzomib was associated with a low rate of typically mild to moderate, non–dose-limiting peripheral neuropathy (PN) and can be administered to patients with either 1) a history of or 2) pre-existing neuropathy without increased risk of PN. Additionally, treatment-emergent renal events resulting in carfilzomib discontinuations were uncommon and there was no association of worsening renal function due to carfilzomib treatment. Finally, hematologic AEs, although common with carfilzomib treatment, were infrequently dose-limiting.

Based on the results of the phase 2 trial PX-171-003-A1, carfilzomib was recently approved by the US Food and Drug Administration for the treatment of relapsed and refractory MM. Herein we describe the design and rationale of the phase 3 randomized study, FOCUS (CarFilzOmib for AdvanCed Refractory MUltiple Myeloma European Study, EudraCT No. 2009-016840-38), which is expected to provide important information to facilitate regulatory approvals around the world and expand treatment options to meet the unmet need of patients with advanced MM disease.

## Methods/design

FOCUS is a phase 3, randomized, open-label, multicenter study comparing 2 treatment regimens for patients with MM for whom no approved alternative therapies are available.

### Objectives

The primary objective of the study is to compare OS in patients with relapsed and refractory MM shown to be refractory to their most recent therapy and who have received all standard approved therapies, randomized to receive carfilzomib or a defined BSC regimen.

Secondary endpoints include PFS, ORR [stringent complete response (sCR) + complete response (CR) + very good partial response (VGPR) + partial response (PR)], CBR (ORR + MR), disease control rate (DCR; CBR + stable disease lasting ≥8 weeks)**,** DOR (calculated separately for overall response, CBR, and disease control endpoints), and safety.

### Patients

Eligible patients must be at least 18 years of age and have measurable MM (defined by serum M protein ≥0.5 g/dL or serum quantitative immunoglobulin A ≥750 mg/dL in patients with IgA secretory MM and/or urine Bence Jones protein ≥200 mg/ 24 h), an Eastern Cooperative Oncology Group (ECOG) Performance Status of 0–2, and a life expectancy of at least 1 month. Patients must have received 3 or more prior therapeutic regimens, including treatment with bortezomib (defined as ≥4 cycles at full dose if tolerated); must have been responsive to at least 1 line of therapy (defined as a ≥25% decrease in M protein or total protein) and have relapsed disease, defined as progression while on or after at least 1 treatment regimen; and must have been refractory to their most recent therapy, defined as nonresponsive (eg, stable disease only, or progressive disease while on treatment), or disease progression within 60 days of discontinuation from therapy. Prior treatments must have included an immunomodulatory agent, an alkylating agent, and a corticosteroid. Additionally, patients must have adequate hepatic function and creatinine clearance of ≥15 mL/min.

Exclusion criteria include: Waldenström’s macroglobulinemia or IgM myeloma, POEMS (polyneuropathy, organomegaly, endocrinopathy, monoclonal gammopathy, and skin changes) syndrome or plasma cell leukemia; major surgery within 21 days prior to randomization; myocardial infarction in previous 3 months, New York Heart Association Class III/IV congestive heart failure; known human immunodeficiency virus seropositivity or active hepatitis; significant neuropathy (Grade ≥3 or Grade 2 with pain) at randomization; other malignancy in past 3 years; and prior carfilzomib treatment.

### Study design and treatment

The study will be conducted in accordance with the Declaration of Helsinki, the International Conference on Harmonization E6, and any applicable regulatory requirements. The protocol and amendments have been reviewed by an Independent Ethics Committee. Approval has been provided by the Ethics Committee for each institution or city where the clinical trial is being held. Written informed consent will be obtained before any protocol-specific tests or procedures are conducted. Estimated sample size is 302 patients, and randomization will be stratified based on number of previous therapies and geographic location (Europe vs non-Europe). Eligible patients will be randomized in a 1:1 ratio to carfilzomib or BSC regimen (Table 1). BSC will initiate on Cycle 1 Day 1 and will include corticosteroid treatment (prednisolone 30 mg every other day, dexamethasone 6 mg every other day, or equivalent), with optional cyclophosphamide 50 mg PO once daily.

**Table 1:** Study schema

**Eligibility criteria**	**Randomization**	**Treatment arms**	**Follow-up**
- Measurable multiple myeloma	Randomized 1:1	**Carfilzomib**	- Confirmed disease progression
- ECOG performance status 0-2	Stratified based on:	*Cycle 1:*	- Long-term follow-up assessments every 8 weeks until death or study closure
- ≥3 prior therapeutic regimens	- 3 vs 4 vs ≥ 5 therapies	20 mg/m^2^ IV D 1,2	
- Responsive to ≥1 line of therapy	- Europe vs non-Europe	27 mg/m^2^ IV D 8, 9, 15, 16	
- Relapsed while on or after 1 therapy		*Cycle 2-9:*	
- Refractory to most recent regimen		27 mg/m^2^ IV D 1, 2, 8, 9, 15, 16	
		*Cycle ≥10:*	
		27 mg/m^2^ IV D 1, 2, 15, 16	
		**Best Supportive Care Regimen**	
		Prednisolone 30 mg QOD	
		OR	
		Dexamethasone 6 mg PO QOD	
		plus optional	
		Cyclophosphamide 50 mg PO QD	
		(max 1400 mg/cycle)	

D, day; ECOG, Eastern cooperative oncology group; IV, intravenous; PO, oral; QD, every day; QOD every other day.

Carfilzomib treatment will be given in 28-day cycles at 20 mg/m^2^ IV on Days 1 and 2 of Cycle 1, escalating to 27 mg/m^2^ IV for Days 8, 9, 15, and 16 of Cycle 1 and continuing at 27 mg/m^2^ for Cycles 2 through 9. For Cycles 10 and higher, carfilzomib will be given at 27 mg/m^2^ IV on Days 1, 2, 15, and 16. Oral hydration, IV hydration, and dexamethasone 4 mg PO or IV during Cycle 1 will be included in the treatment regimen. Dosing will be modified as necessary (20 mg/m^2^, 15 mg/m^2^, or 11 mg/m^2^) to manage toxicity.

For all patients randomized to carfilzomib, required concomitant medications during Cycle 1 include dexamethasone (4 mg PO or IV) and ciprofloxacin (500 mg PO QD). Valacyclovir or an equivalent antiviral is also required for patients treated with carfilzomib who have a history of herpes zoster. Additional medications that are permitted include bisphosphonates and other supportive care therapies at the investigator’s discretion. Uric acid-lowering agents, red blood cell and platelet transfusions, erythropoiesis-stimulating agents, and approved growth factors may be given when clinically indicated. Concurrent treatment with any anticancer therapy or radiation to large marrow reserves with therapeutic or palliative intent are not permitted, nor are corticosteroids at doses higher than those stated in the protocol. Plasmapheresis is not permitted.

Patients will continue randomized study treatment until confirmed disease progression (progressive disease, PD) or unacceptable toxicity. Following confirmation of PD or discontinuation from study treatment, all patients will enter long-term follow-up (LTFU) for survival. Crossover is not allowed upon progression.

### Assessment of efficacy

Patients will be evaluated for disease response and progression according to the International Myeloma Working Group (IMWG) response criteria [25] and for MR according to the European Group for Blood and Marrow Transplantation (EBMT) criteria [26,27]. Evaluations will occur on Day 1 of each cycle, at the post-treatment visit, and during LTFU for those who did not have PD prior to discontinuing treatment. PD must be confirmed by 1) 2 consecutive measurements of rising serum or urine M-protein, 2) development of new lesions or definite increase in size of existing bone lesions or soft tissue plasmacytomas, or 3) hypercalcemia attributed solely to MM. Tumor response for all categories must be confirmed by 1) 2 consecutive M-protein measurements, 2) plasmacytoma evaluation where relevant, 3) no evidence of new or progressive bone lesions and 4) bone marrow biopsy for CR/sCR.

### Assessment of safety

All patients who receive at least 1 dose of any study-specific treatment will be included in the safety analysis. Adverse event (AE) data will be collected at each visit from the time the patient signs the consent form and will be coded using the Medical Dictionary for Regulatory Activities (MedDRA) and assigned a severity grade using the National Cancer Institute-Common Terminology Criteria for Adverse Events (NCI-CTCAE) v4.0 grading scale. The investigator may discontinue study treatments for the following reasons: patient withdrawal of consent, confirmed PD, unacceptable toxicity, noncompliance with study procedures, requirement for alternative therapy, and intercurrent illness or worsening of a chronic condition; however, patients will be encouraged to remain on study for survival assessment. Patients with a dose reduction due to toxicity will remain on study; if the reduced dose is well tolerated, the patient may be rechallenged with the original dose at the start of the next cycle.

### Statistical analysis

The primary endpoint of the study is OS, defined as the time from randomization to death due to any cause. The study will provide 80% power to detect a 30% reduction in the risk of death. The primary inferential comparison for OS between treatment groups will use the log-rank test assessed against an overall 1-sided significance level of 0.025 stratified by the randomization stratification factors (number of prior therapies and geographical location). The hazard ratio for treatment group will be estimated using a stratified Cox proportional hazards model. The distribution of OS times will be summarized using the Kaplan-Meier method.

An independent data monitoring committee (DMC) has been convened for this study and will act in an advisory capacity in safeguarding the interests of study patients, assessing interim safety and efficacy and for monitoring the overall conduct of the trial.

The inferential tests associated with secondary endpoints PFS, ORR, CBR, DCR, and DOR (including duration of clinical benefit and duration of disease control) will be performed in accordance with a closed testing procedure (CTP) to adjust for the multiplicity. Under the CTP, if the null hypothesis for the primary endpoint (OS) is rejected at the 1-sided significance level of 0.025, then testing of the secondary endpoints will proceed in a sequential step-down manner and a 1-sided significance level of 0.025 will be used at each testing step. Testing of secondary endpoints will continue provided the null hypothesis for each of the previously tested secondary endpoints is rejected.

The distribution of PFS times will be summarized descriptively using the Kaplan-Meier method. The inferential comparison between treatment groups will use the log-rank test stratified by the randomization stratification factor. The hazard ratio for treatment group will be estimated using a stratified Cox proportional hazards model. The inferential comparison between treatment groups for the ORR, CBR, and DCR endpoints will be made using the Cochran-Mantel-Haenszel chi-square test, stratified by the randomization stratification factor. The odds ratio to measure the relative treatment effect and the 95% CI will be estimated using the Mantel-Haenszel method. The DOR will be calculated for patients who achieve sCR, CR, VGPR, and PR. The inferential comparison between treatment groups will use the log-rank test stratified by the randomization stratification factors (region: Europe vs non-Europe, and number of prior lines of therapy: 3 vs 4 vs ≥5). The duration of overall response will be summarized descriptively using the Kaplan-Meier method.

The comparisons for the primary and secondary efficacy endpoints will be between the 2 randomized treatments groups (Regimen C vs Regimen BSC) based on the intent-to-treat population (ITT), consisting of all randomized patients.

### Status

FOCUS began enrolling patients in September 2010 with a target enrolment of 302 patients. Approximately 100 institutions are being recruited to participate. The study is currently accruing patients in 7 countries and over 40 sites throughout Europe and is expanding to other parts of the world, with 211 patients enrolled as of the end of June 2012.

## Discussion

Based on results of previous trials with similar carfilzomib treatment, carfilzomib is anticipated to provide prolonged disease control compared with BSC [16-18,28]. This phase 3 trial will provide more rigorous data compared with those already available for carfilzomib, as it will not be confounded by crossover and is the first carfilzomib study with OS as the primary endpoint. Additionally, the larger study group will provide more robust secondary response data, including PFS and ORR. It has been demonstrated that maximal response is associated with better outcome in a variety of different treatment settings [6]. In light of this current view of the importance of attaining CR in MM, more robust data will be beneficial.

Previous studies have reported good tolerability for carfilzomib [22-24]. Carfilzomib has shown specific long-term tolerability in follow-up of the pivotal 171-003-A1 trial, as well as an extension trial in which patients who completed other carfilzomib studies are eligible to enroll (PX-171-010, NCT00884312) [17,29]. Additionally, an ongoing phase 1/2 study has shown carfilzomib to be tolerable at doses up to 56 mg/m^2^[18]. The present study will expand upon the existing carfilzomib single-agent tolerability data set.

This is a study of heavily pretreated patients and, similar to studies with other newer drugs in relapsed and refractory MM, challenges may arise in the interpretation of the results. A recent study by the IMWG confirmed that patients generally have a poor outcome after becoming refractory to currently available therapies [7]. The IMWG data establish a relevant context for interpreting the results of studies involving novel drugs and provide information regarding the natural history of relapsed MM. This information will be useful in interpreting the results of the present study.

With respect to the study design, the European Medicines Agency (EMEA) strongly recommended evaluating whether carfilzomib increased survival in heavily-pretreated patients with MM, necessitating the inclusion of a BSC comparator arm. This ultimately proved to be both scientifically and ethically challenging. In particular, the identification of a universally acceptable salvage regimen for treatment of patients with advanced MM is quite difficult as treatment options for patients with relapsed and refractory MM are frequently limited due to concurrent disease or patients’ inability to tolerate conventional therapies. Several meetings with European experts in MM were held with the expressed aim of defining an acceptable control arm for this trial. While a number of individuals suggested testing carfilzomib against an active control arm, the majority of experts favored a control arm permitting the use of optional cyclophosphamide as it has been shown that continuous low-dose oral cyclophosphamide in combination with prednisone has resulted in response rates of 41–69% with OS up to 28.6 months [30-32]. Following careful thought and consideration of the consensus view, the lead investigators concluded that the best approach to address the needs of both patients and the recommendation of the regulatory agency was for the control arm to include a standard regimen of dexamethasone (or equivalent dosing of a comparable glucocorticoid) with the option of adding continuous low-dose oral cyclophosphamide.

To address the potential ethical concerns raised by the trial design, an extension study is planned that will offer treatment with carfilzomib to control-arm patients who complete the study, provided that an interim analysis of the study data demonstrates a statistically significant survival benefit for carfilzomib relative to BSC. This option would also permit patients progressing on BSC to cross over to the carfilzomib treatment arm. Furthermore, patients are not restricted from receiving further anti-myeloma therapy following study drug discontinuation.

In conclusion, the results of FOCUS in patients with relapsed and refractory MM will provide important information to facilitate regulatory approvals around the world and expand treatment options to meet the unmet need of these patients with advanced disease.

## Competing interests

RH has no competing interests to declare. RB declares that he is a current employee of Onyx Pharmaceuticals Inc., South San Francisco, CA and has stock ownership in Onyx. SR declares that she is a current employee of Onyx Pharmaceuticals Inc., South San Francisco, CA and has stock ownership in Onyx. BK declares that she is a current employee of Onyx Pharmaceuticals Inc., South San Francisco, CA, has stock ownership in Onyx and has received a salary from Onyx for a patent related to the manuscript. HL has no competing interests to declare.

## Authors' contributions

RH has made substantial contributions to conception and design, or acquisition of data, or analysis and interpretation of data and was involved in drafting/revising the manuscript. RB has made substantial contributions to conception and design, or acquisition of data, or analysis and interpretation of data and was involved in drafting/revising the manuscript. SR has made substantial contributions to conception and design, or acquisition of data, or analysis and interpretation of data. BK has made substantial contributions to conception and design, or acquisition of data, or analysis and interpretation of data and was involved in drafting/revising the manuscript. HL has made substantial contributions to conception and design, or acquisition of data, or analysis and interpretation of data and was involved in drafting/revising the manuscript. All authors read and approved the final manuscript.

## Pre-publication history

The pre-publication history for this paper can be accessed here:

http://www.biomedcentral.com/1471-2407/12/415/prepub
